# The influence of two neurohormones on breast cancer: prolactin and melatonin

**DOI:** 10.1590/1806-9282.20251014

**Published:** 2025-12-05

**Authors:** Cecília da Silva Ferreira, Kátia Cândido Carvalho, Isaque da Silva Ferreira, Giovanna Santos Cavalcanti, José Cipolla, Pedro Egydio Oliveira Zambotti, Edmund Chada Baracat, José Maria Soares

**Affiliations:** 1 Laboratório de Ginecologia Estrutural e Molecular, Disciplina de Ginecologia, Departamento de Obstetrícia e Ginecologia, Hospital das Clínicas, Faculdade de Medicina, Universidade de São Paulo – São Paulo (SP), Brazil.; 2 Universidade de São Paulo, Institute of Biomedical Sciences, Department of Physiology and Biophysics – São Paulo (SP), Brazil.

## INTRODUCTION

The influence of prolactin (PRL) and melatonin on breast cancer represents a complex interplay of hormonal signaling with significant implications for tumor progression and therapeutic strategies. PRL, a polypeptide hormone produced primarily by the pituitary gland but also locally in mammary tissue, plays a dual role in breast physiology and pathology^1^. It acts synergistically with estrogen and progesterone to promote proliferation and differentiation of mammary epithelial cells, processes that are essential for lactation^1,2^. However, its involvement in breast cancer remains debated, with most studies suggesting proliferative and pro-metastatic effects. PRL exerts its actions through specific transmembrane receptors (prolactin receptor [PRLR])^1,2^, activating signaling pathways such as JAK2/STAT5, Ras/MAPK, and PI3K/AKT, which drive cell survival, proliferation, and differentiation. Controversially, while PRL overexpression in cell culture enhances tumor growth, PRLR knockdown or antagonism can lead to more aggressive but less differentiated tumors, highlighting the context-dependent nature of PRL's role in breast cancer. Therefore, blood PRL concentration is important for the development of cancer^1-4^.

Melatonin, a neurohormone secreted by the pineal gland, exhibits oncostatic properties that counterbalance PRL's oncogenic effects. Its pleiotropic actions include modulation of cell proliferation, apoptosis, angiogenesis, and immune response, making it a promising candidate for breast cancer therapy. Melatonin operates through receptor-dependent (MT1/MT2) and receptor-independent mechanisms. By binding to MT1/MT2 receptors, it downregulates estrogen receptor alpha (ERα) activity and aromatase expression, reducing estrogen-driven tumor growth^5,6^. Independent of receptors, melatonin's antioxidant properties scavenge reactive oxygen species (ROS), protecting against DNA damage and suppressing tumor initiation. Additionally, melatonin inhibits angiogenesis by reducing vascular endothelial growth factor (VEGF) and hypoxia-inducible factor 1 (HIF-1) expression and induces apoptosis via mitochondrial dysfunction and caspase activation. Clinical evidence supports its role in reducing Ki-67(+) cells in estrogen receptor-positive (ER+) tumors and synergizing with chemotherapy to inhibit metastasis^5,6^.

Emerging evidence suggests that melatonin's regulatory influence on PRL levels may critically impact breast cancer pathogenesis^5,6^, particularly in hyperprolactinemic conditions^7^. This review evaluates the interplay between melatonin and PRL signaling in mammary oncogenesis, with particular emphasis on melatonin's potential to modulate PRL-driven tumor progression through endocrine and paracrine mechanisms.

## METHODS

### Identification of studies

A narrative review search was conducted using the databases PubMed, SciELO, Google Scholar, and LILACS from January 2000 to November 2024. The search employed MeSH terms (Prolactin, Melatonin, Breast Cancer) combined with the Boolean operator "AND" to ensure relevance. Only full-text articles published in English, French, Spanish, or Portuguese were considered.

### Quality assessment

The methodological quality of included studies was evaluated using preclinical studies: SYRCLE's risk-of-bias tool (for animal research) to assess randomization, blinding, and outcome reporting; clinical studies: Newcastle-Ottawa Scale (NOS) for observational studies, focusing on selection, comparability, and outcome validity; and in vitro studies: modified grade criteria for reproducibility, controls, and statistical robustness. The low score of method quality was not included in this review.

### Study selection

Two independent reviewers screened titles and abstracts for eligibility. Inclusion criteria focused on experimental, preclinical, and clinical studies investigating the roles of PRL and melatonin in breast cancer. Exclusion criteria encompassed non-relevant studies, unavailable full texts, and non-English/Portuguese/Spanish/French publications. A total of 40 articles were selected based on relevance, methodological rigor, and publication date, with discrepancies resolved through consensus or consultation with a third reviewer.

### Data extraction and analysis

Data were extracted and organized into structured tables, including study design, sample characteristics, hormonal mechanisms, and key findings. A narrative synthesis was performed to compare results across studies, identifying patterns in melatonin's oncostatic effects and PRL's oncogenic role. Mechanistic insights (e.g., JAK/STAT signaling and antioxidant pathways) were prioritized for interpretation.

## RESULTS

Extensive evidence demonstrates that PRL critically regulates mammary tissue dynamics, influencing both developmental and maturational processes in coordination with estrogen and progesterone. Although the roles of estrogen and progesterone in breast tumorigenesis are well characterized, PRL's contribution remains controversial. Compelling data indicate that PRL drives oncogenic processes, including cellular proliferation, survival, motility, and vascularization in breast cancer^8^. Notably, clinical studies associate the long isoform of the PRLR with increased metastatic potential and worse clinical outcomes^9^, suggesting its utility as a prognostic biomarker^10^.

Experimental evidence supports the mitogenic role of PRL, as PRL-overexpressing MDA-MB-435 cells exhibit increased proliferation, tumor growth, migration, and metastasis. However, conflicting data indicate that PRLR-deficient breast cancer cells display poorer differentiation and heightened aggressiveness, whereas PRL treatment suppresses tumor growth in certain models^11^.

PRLR activation initiates multiple oncogenic signaling cascades, with the JAK2–STAT5 pathway playing a particularly well-characterized role in mammary tumorigenesis^12,13^. Notably, PRL demonstrates ligand-independent activation of ERα and stimulates parallel pathways including PI3K/AKT and MAPK, suggesting broad mechanistic involvement in breast cancer progression^14^. This signaling network is further amplified through synergistic interactions with estradiol (E2), creating potential therapeutic vulnerabilities. Of particular clinical relevance, the Ras/Raf/MAPK axis appears preferentially activated in aggressive tumor subtypes^15^, highlighting its importance as a therapeutic target.

Despite structural and signaling differences, as well as distinct receptor systems, PRL and E2 exhibit functional crosstalk. PRL can activate ERα, while E2 upregulates PRL and PRLR transcription. This synergistic interaction may hold therapeutic implications for breast cancer treatment^16^.

Emerging evidence suggests a link between circulating PRL levels and increased breast cancer risk in ER+/progesterone receptor-positive (PR+) patients; however, the prevailing paradigm attributes PRL's oncogenic effects predominantly to local autocrine/paracrine actions rather than systemic endocrine mechanisms. Experimental models show that PRL stimulates MCF-7 breast cancer cell proliferation through autocrine/paracrine activation of the JAK/STAT/cyclin D1 pathway. This local proliferative effect appears particularly relevant in ER+/PR+ tumors, where PRL cooperates with E2 to drive oncogenesis, as shown in transgenic mouse models developing ER+ tumors under conditions of elevated PRL expression^17-19^.

PRL contributes to mammary tumorigenesis through multiple mechanisms, including modulation of apoptosis and angiogenesis. Experimental evidence demonstrates that PRL antagonists upregulate apoptotic genes^20^, while prolonged PRLR knockdown increases cell death and reduces metastatic potential in breast cancer cell lines^21^. Additionally, PRL promotes tumor progression through proangiogenic effects, stimulating neovascularization and endothelial cell migration^19^. Interestingly, PRL can be proteolytically cleaved to form vasoinhibins, which counterbalance these effects through their antiangiogenic properties^22,23^, highlighting the complex dual role of PRL in tumor vascularization.

PRL exerts pleiotropic effects that extend beyond epithelial cells to critically modulate the breast tumor microenvironment. Emerging evidence demonstrates PRL's capacity to regulate immune cell function and cytokine production, establishing a pro-tumorigenic niche^24^. Furthermore, PRL directly influences extracellular matrix (ECM) remodeling and increases mammary tissue density–both established risk factors for malignant transformation^25^. Significantly, PRL enhances cellular motility through cytoskeletal reorganization, providing a mechanistic basis for its well-documented role in metastatic progression^10^.

While epidemiological studies associate elevated serum PRL levels with increased ER+ breast cancer risk^26^, gene expression analyses reveal PRLR presence across ER+, HER2+, and triple-negative tumors, challenging this paradigm. The prognostic significance of PRLR remains controversial, with some studies linking high expression to poor outcomes and others to better-differentiated tumors—discrepancies potentially arising from transcript- vs. protein-level differences^8^. Experimental data are similarly conflicting, possibly due to variability in models, PRL sources, or microenvironmental contexts. Clarifying PRL/PRLR roles in breast cancer will require advanced studies, including genetically engineered models for more precise investigation^27^.

Given the established oncogenic role of PRL signaling in mammary carcinogenesis, melatonin represents a promising therapeutic candidate through its dual regulation of PRL secretion and ER activity^5-8^. As a pleiotropic hormone, melatonin negatively modulates multiple reproductive hormone pathways–including growth hormone (GH), E2, luteinizing hormone (LH), and PRL–that collectively drive breast neoplasia^28-31^. Mechanistically, melatonin's antiproliferative effects are evidenced by significant reductions in Ki-67+ and PCNA+ tumor cells^32^, underscoring its potential as both a preventive and therapeutic agent in hormone-responsive breast cancers.

Melatonin mediates its oncostatic effects in breast cancer through dual mechanisms: (1) receptor-dependent pathways involving MT1/MT2-mediated modulation of nuclear transcription factors and calmodulin-dependent signaling and (2) receptor-independent antioxidant activity^33,34^. Notably, melatonin also demonstrates potent antiangiogenic properties by downregulating VEGF and HIF-1 expression, effectively suppressing tumor vascularization^34^.

Melatonin induces tumor cell apoptosis through mitochondrial dysfunction, triggering ROS accumulation and cytochrome C release while upregulating Bax and activating caspases^29,35^. Intriguingly, these pro-apoptotic effects may occur independently of melatonin receptors or antioxidant activity^36^, though context-dependent variations in cell death responses have been reported across cancer types^37^, warranting further mechanistic investigation.

Melatonin's antimetastatic action on breast cancer has been demonstrated through its modulation of the expression of adhesion and invasion molecules, such as ROCK-1^38^. Another well-described and significant property of melatonin in combating cancer development is its immunomodulatory effect. Melatonin can stimulate lymphocytes, monocytes, macrophages, and natural killer (NK) cells, thereby increasing cytokine production^38^. Lastly, melatonin can suppress telomerase activity in breast cancer cells, further reinforcing its multiple oncostatic activities^35^.

### Melatonin and prolactin on breast cancer context

**Figure 1:**
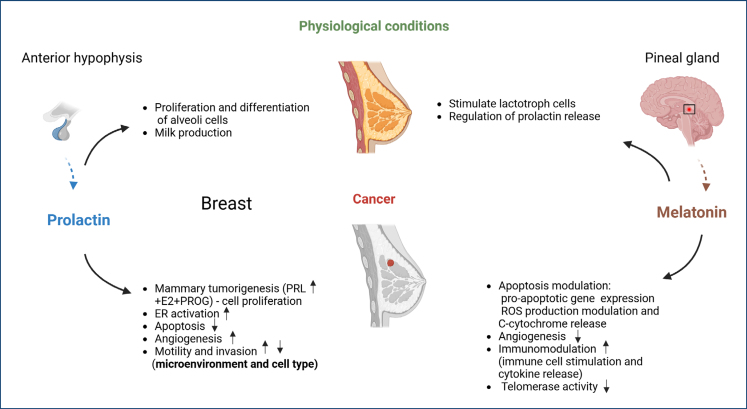
Prolactin and melatonin's impacts on breast cancer. Created using BioRender.com.

Melatonin's regulation of PRL secretion represents a critical therapeutic axis in PRL-driven breast cancers. By downregulating ERα (which PRL can activate ligand-independently) and suppressing aromatase-mediated E2 production, melatonin counteracts PRL's oncogenic signaling (Figure 1). However, the melatonin–PRL interplay remains incompletely characterized, necessitating advanced genetic models to elucidate mechanistic interactions and develop targeted therapies.

## DISCUSSION

This narrative review synthesizes compelling evidence on the antagonistic roles of PRL and melatonin in breast cancer pathogenesis and progression. PRL promotes tumorigenesis through JAK/STAT, MAPK, and PI3K/AKT signaling, enhancing proliferation, angiogenesis, and immune evasion, particularly in ER+ subtypes. Conversely, melatonin exhibits oncostatic effects by suppressing PRL secretion, scavenging ROS, inhibiting VEGF-mediated angiogenesis, and inducing apoptosis via mitochondrial pathways^12-31,39,40^. Notably, melatonin's downregulation of ERα and aromatase activity further disrupt hormone-driven tumor growth. Clinical correlations link elevated PRL to poorer outcomes in ER+ breast cancer, while preclinical models demonstrate melatonin's potential to synergize with chemotherapy and reduce metastasis. However, heterogeneity in study designs and a paucity of large-scale clinical trials underscore the need for standardized research to translate these findings into therapeutic strategies. Collectively, these insights position the melatonin–PRL axis as a promising target for precision oncology in breast cancer^22-40^.

The strength of this review highlights the dual role of PRL in promoting breast cancer progression and melatonin's counteractive oncostatic effects through multiple mechanisms, including PRL suppression, antioxidant activity, and immune modulation. While the study benefits from a comprehensive analysis of recent preclinical and clinical evidence, limitations include heterogeneity in study designs and the lack of large-scale human trials to confirm translational potential. Nevertheless, the robust mechanistic data and emerging clinical observations position melatonin as a promising adjuvant therapy, particularly for hormone-responsive breast cancers, warranting further investigation into optimal dosing and combination strategies.

## CONCLUSION

Emerging evidence highlights melatonin as a potent oncostatic agent in breast cancer, countering PRL-driven oncogenesis through direct hormonal modulation, antioxidant activity, and immune regulation. While preclinical studies underscore its therapeutic potential, clinical translation requires further mechanistic exploration—particularly in PRL-sensitive subtypes—and robust trials to validate efficacy. Targeting the melatonin–PRL axis offers a promising, low-toxicity strategy to augment current therapies, urging interdisciplinary research to harness its full clinical potential.

## Data Availability

The datasets generated and/or analyzed during the current study are available from the corresponding author upon reasonable request.
